# Longitudinal Follow Up of Patients Colonized with Clostridioides difficile: a Retrospective Cohort Study

**DOI:** 10.1017/ash.2024.190

**Published:** 2024-09-16

**Authors:** Bianca Bilodeau-Harnois, Yves Longtin, Leighanne Parkes

**Affiliations:** McGill University; Jewish General Hospital

## Abstract

**Background:** Patients colonized with Clostridioides difficile are at risk of transmitting C. difficile to other patients, and of developing C. difficile infection (CDI). Known risk factors for carriage include previous hospitalization, gastric acid suppression and previous CDI. Data regarding duration of carriage and its predictors are lacking but could be useful to better understand the natural evolution of carriage and better estimate the likelihood of transmission or progression to CDI. **Methods:** We performed a retrospective cohort study of C. difficile colonized patients with > = 1 admission to a tertiary academic institution between November 2013 and January 2017. Colonization status was determined upon hospital admission by detection of TcdB gene by polymerase chain reaction on a rectal screening swab, as part of a systematic screening program. Overall duration of carriage and predictors of prolonged carriage were explored using Kaplan-Meier methods and Cox regression. **Results:** There were 134 patients, who after having a positive initial screening test (and therefore identified as colonized with C. difficile), had subsequent testing. The median age was 77 years (IQR, 66 to 85), and 53.6% of the patients were female. After hospital discharge, 26 (19.4%) colonized patients progressed to CDI. Mean duration of follow up was 269 days, with a median of 179 days. Median duration of carriage was 211 days, (95% confidence interval (CI) [157, 264]). Predictors associated with decreased duration of C. difficile colonization included younger age (HR per unit decrease (year), 1.013; 95% CI, 1.025 to 1.001; p=0.03), and receipt of antibiotics in the 3 months prior to first admission (mean days to clearance of patients with and without recent antibiotic use, 252 days vs 372 days, respectively; HR, 1.55; 95% CI, 1.01 to 2.36; p < 0 .04). By contrast, the presence of comorbidities (e.g. heart failure, diabetes, cancer, and chronic kidney disease), the use of proton-pump inhibitors (PPIs), the receipt of antibiotics during the first admission, and the duration of first hospitalization were not associated with significant differences in duration of carriage. **Conclusion:** This study is the largest cohort of C. difficile carriers with longitudinal follow up of their colonization status. It highlights the extended duration of carrier status especially in older patients and identifies predictors of prolonged carriage. Further studies are needed to understand the underlying relationship with the predictors identified in this study.

